# Measurement of Magnesium, Zinc, and Copper in Human Serum by Using Isotope Dilution Inductively Coupled Plasma Mass Spectrometry (ID ICP-MS)

**DOI:** 10.1155/2023/6612672

**Published:** 2023-10-09

**Authors:** Jian Yang, Lixia Chi, Shengmin Li

**Affiliations:** ^1^Institute of Disaster Prevention, Langfang 065201, China; ^2^Beijing Institute of Medical Device Testing, Beijing 101111, China

## Abstract

In order to evaluate the reliability of the ID ICP-MS method for the measurement of magnesium, zinc, and copper in human serum, we investigated the traceability, precision, trueness, and uncertainty of the method. This method traces the contents of magnesium, zinc, and copper in human serum to the standard materials NIST SRM3131a, SRM3168a, and SRM3114 respectively, thus completing the traceability to SI unit. The repeatability of this method for measuring magnesium, zinc, and copper in the human serum reference material GBW09152 was found to be 0.2%, 0.7%, and 0.6% (*n* = 9), respectively. The measurement, when employed to measure the magnesium, zinc, and copper in standard materials, had caused a maximum deviation of less than 0.88%, 1.35%, and 1.15%, respectively. The measurement results are within the stated uncertainty range of standard materials. The expanded uncertainties were 0.2 mg·kg^−1^, 0.04 mg·kg^−1^, and 0.08 mg·kg^−1^ (*K* = 2) for magnesium, zinc, and copper, respectively. Therefore, this method has high trueness, good reproducibility, and simple operation and is suitable for tracing the values of magnesium, zinc, and copper in human serum.

## 1. Introduction

Isotope dilution mass spectrometry, which has many advantages over conventional methods, is the only authoritative method that can directly provide trace and ultratrace values [1]. In common analysis methods, the accuracy of the results may be affected by the loss of the elements to be tested in the process of sample pretreatment, matrix effects, and instrument signal drift [2–5]. Isotope dilution mass spectrometry is the method which measures only the isotope abundance ratio in the sample. The abundance ratio becomes a constant value when the concentration of the isotopic spike is added to the sample and reaches the equilibrium with the absence of external contamination, and the sample loss in the process of sample separation and concentration does not affect the abundance ratio [6]. Isotope dilution method can greatly eliminate interference and errors caused by sample pretreatment and has thus been confirmed by the Consultative Committee for Amount of Substance: Metrology in Chemistry and Biology (CCQM) as one of the five methods with absolute measurement properties [7, 8]. This isotope dilution mass spectrometry originated from the UK Government Chemist Laboratory (GCL) [9, 10]. We have established isotope dilution mass spectrometry for the detection of potassium and selenium in human serum using this principle [11]. Compared with conventional isotope dilution mass spectrometry, it has the advantage of not having to calibrate the concentration of the isotopic spike during the standardization process. It can determine the chemical composition in serum only by means of measuring the selected pair of isotope abundance ratios, thus reducing the requirements on the performance of the mass spectrometry instrument [12–17].

Trace elements of magnesium, zinc, and copper in the human body are closely related to people's health [18–22]. Currently, there is no reference method for measuring zinc and copper in serum by using isotope dilution mass spectrometry in the Joint Committee on Traceability on Laboratory Medicine (JCTLM) list. Only the measurement of the concentration of magnesium in serum by using traditional isotope dilution mass spectrometry is available [23–25]. A precise method for analyzing magnesium, zinc, and copper in human serum by using two-step isotope dilution mass spectrometry was established in this laboratory. The repeatability of this method for measuring magnesium, zinc, and copper in the human serum reference material GBW09152 was found to be 0.2%, 0.7%, and 0.6% (*n* = 9), respectively. The expanded uncertainties were 0.2 mg·kg^−1^, 0.04 mg·kg^−1^, and 0.08 mg·kg^−1^ (*K* = 2) for magnesium, zinc, and copper, respectively. With the quality of easy operation, high trueness, and good reproducibility, this method is suitable as a reference method for measuring magnesium, zinc, and copper elements in human serum.

## 2. Materials and Methods

### 2.1. Materials and Reagents

The laboratory is a class 100,000 cleanroom. The experimental water was provided by a water purification system: Milli-Q Advantage (Millipore, USA). The nitric acid (Ultrapure-BVIII) and hydrochloric acid (MOS grade) used in the experiment were produced by the Beijing Institute of Chemical Reagents, China. The microanalytical balance used for sample weighing was Mettler Toledo XS205 (Switzerland), and the sample analysis was performed on an ICP mass spectrometer: ELAN DRC-e (PerkinElmer, USA). The standard materials used for method tracing were magnesium (Mg) standard solution SRM3131a (NIST, USA), copper (Cu) standard solution SRM3114 (NIST, USA), and zinc (Zn) standard solution SRM3168a (NIST, USA). The isotope spikes used in the method were ^25^Mg (assay: 97%), ^65^Cu (assay: 99%), and ^67^Zn (assay: 94%), concentrated isotopes from the Oak Ridge National Laboratory, USA. Trueness verification was performed by using inorganic components in frozen human serum GBW09152 (National Institute of Metrology, China), ERM-DA120a (LGC, UK) and electrolytes in frozen human serum SRM956d (NIST, USA).

### 2.2. Instrument Parameters

The instrument parameters for this research are listed in Table 1.

### 2.3. Methods

(1) A two-step dilution of NIST SRM3131a was performed by using the weighing method in a ratio of approximately 1 : 20, and a three-step dilution of NIST SRM3168a and NIST SRM3114 in a ratio of approximately 1 : 20. The final dilution must be prepared on the day of the experiment.
(2) The concentrated isotopic metal chips of ^25^Mg, ^65^C, and ^67^Zn were dissolved with BVIII grade nitric acid and diluted to an appropriate concentration with ultrapure water, followed by a two-step dilution on the day of the experiment.
(3) A mixed solution of magnesium standard solution and ^25^Mg isotope diluent, a mixed solution of zinc standard solution and ^67^Zn isotope diluent, and a mixed solution of copper standard solution and ^65^Cu isotope diluent were prepared, respectively, by using the weighing method. A mixed solution of the serum sample and ^25^Mg isotope diluent, a mixed solution of the serum sample and ^67^Zn isotope diluent, and a mixed solution of the serum sample and ^65^Cu isotope diluent were also prepared, respectively, by using the weighing method. The isotopic ratio (^24^Mg/^25^Mg or ^66^Zn/^67^Zn or ^63^Cu/^65^Cu) in the mixture solution was close to 1, and at the same time, the cps signal intensities of the corresponding isotopes in the mixture of the standard solution and the isotope diluent as well as in the mixture of the serum sample and the isotope diluent were close.
(4) Solutions of magnesium, copper, and zinc with appropriate concentrations were prepared on the day of the experiment so that the signal intensity of ^24^Mg, ^66^Zn, and ^63^Cu in the solution is consistent with that of the corresponding isotopes in the mixed solution in (3).
(5) Solutions of ^25^Mg, ^67^Zn, and ^65^Cu diluents with appropriate concentrations were prepared on the day of the experiment so that the signal intensity of the isotopes in the solution is consistent with that of the corresponding isotopes in the mixed solution in (3).
(6) Mass spectrometric procedures: the concentrations of magnesium, zinc, and copper elements in the serum sample were calculated according to the concentration formula (1) in the isotope dilution mass spectrometry [9]:
  (1) $$\begin{array}{c} C_{x}=C_{z}\cdot \frac{m_{Z_{c}}}{m_{Y_{c}}}\cdot \frac{m_{Y}}{m_{X}}\cdot \frac{R_{Y}-R_{B}}{R_{B}-R_{Z}}\cdot \frac{R_{Z}-R_{B_{c}}}{R_{B_{c}}-R_{Y}}-C_{B}. \end{array}$$

In this formula, C_z_ is the concentration of the standard solution, *m*_*Y*_ is the mass of the enriched isotope added to the serum sample, *m*_*X*_ is the mass of the serum sample added to the mixture of the serum sample and the enriched isotope, m_Yc_ is the mass of the enriched isotope added to the standard solution, and *m*_zc_ is the mass of the standard solution added to the mixture of the standard solution and the enriched isotope. Rz is the isotope ratio of ^24^Mg/^25^Mg or ^66^Zn/^67^Zn or ^63^Cu/^65^Cu in the standard solution, and *R*_*Y*_ is the isotope ratio of ^24^Mg/^25^Mg (^66^Zn/^67^Zn or ^63^Cu/^65^Cu) in the enriched isotope. *R*_*B*_ is the isotope ratio of ^24^Mg/^25^Mg (^66^Zn/^67^Zn or ^63^Cu/^65^Cu) in the mixture of serum sample and the enriched isotope, and *R*_Bc_ the isotope ratio of ^24^Mg/^25^Mg (^66^Zn/^67^Zn or ^63^Cu/^65^Cu) in the mixture of standard solution and the enriched isotope. *C*_*B*_ is a blank in the measurement process.

It was found that the fluctuation of the isotope ratio in the standard solution or the enriched isotope had little impact on the final results, while the measurement fluctuation of the isotope ratio in the mixed solution would lead to changes in the final results. Therefore, the mixed solution of the standard solution and the enriched isotope and the mixed solution of the serum sample and the enriched isotope must be alternately measured six times so as to reduce the error introduced by instrument measurement drift.

## 3. Results and Discussion

### 3.1. Determination of Instrument Measurement Conditions

In this experiment, the dynamic reaction cell mode of inductively coupled plasma mass spectrometry (ICP-MS) was used to eliminate interference. When analyzing magnesium, interference from NaH was severe, and interference from Ca^++^ and LiO may also exist. The condition optimization was aiming to obtain the best analysis effect when analyzing ^24^Mg, and the appropriate flow rate of the reaction gas CH_4_ and argon. When analyzing zinc, interference from SO_2_, ClO_2_, and ArP may exist, and interference on ^67^Zn was more likely to occur. The condition optimization was aimed at obtaining the best analysis effect when analyzing ^67^Zn and the appropriate flow rate of the reaction gas CH_4_ and argon. When analyzing copper, interference from SO_2_ and PO_2_ may exist, while all these interferences can be ignored in actual analysis. Therefore, this experiment analyzed copper in blood under standard mode.

### 3.2. Deduction of Signal Background in the Experimental Method

**Figure 1:**
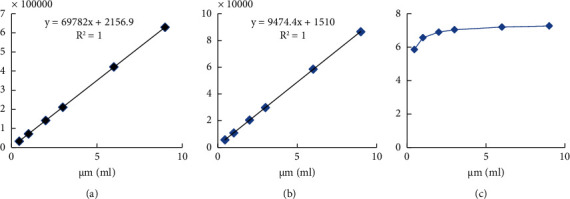
(a) Relationship between the response signal of ^24^Mg and magnesium concentration; (b) relationship between the response signal of ^25^Mg and magnesium concentration; (c) relationship between the ratio of ^24^Mg to ^25^Mg response signals and sample concentration.

Solutions of serum magnesium with 6 concentration gradients ranging from 0.5 *μ*g/ml to 9 *μ*g/ml were measured. The response signals of ^24^Mg and ^25^Mg are linearly related to the concentration range of magnesium (see Figures 1(a) and 1(b)), with a linear correlation coefficient (*r*) of 0.99999. However, the ratio of ^24^Mg response signal to ^25^Mg response signal was not constant but gradually tended to be constant with the increase of concentration (see Figure 1(c)). This is because the straight lines in Figures 1(a) and 1(b) have a nonzero intercept, indicating the presence of a blank response signal for ^24^Mg and ^25^Mg. As the concentration becomes smaller, the relative proportion of the response signal becomes higher, and the impact on the ratio of ^24^Mg to ^25^Mg response signal is more significant. Therefore, this blank response signal cannot be ignored. After subtracting the blank response signal from the measured impact signal, a constant value is obtained for the ratio of the ^24^Mg to ^25^Mg·g response signals. The same is true for the determination of ^66^Zn/^67^Zn and ^63^Cu/^65^Cu, so in this experiment, the response signals used are all values after deducting the blank signal.

### 3.3. Effect of Solution Reaction System on Measurement Precision

It was found in the experiment that using a 0.02% hydrochloric acid system can improve the stability of ^66^Zn/^67^Zn measurement. Therefore, the determination of zinc in serum was indeed carried out in a 0.02% hydrochloric acid system.

### 3.4. Process Blank (LOB) and Detection Limits (LOD)

While preparing the mixture of serum sample and the enriched isotope, an appropriate amount of ^25^Mg (^67^Zn or ^65^Cu) solution was taken into the blank sample tube as a process blank, so that the ^24^Mg/^25^Mg (^66^Zn/^67^Zn or ^63^Cu/^65^Cu) in the process blank was approximately equal to 2. The process blank was determined along with the samples in the same batch. The process blanks for magnesium, zinc, and copper in serum were 0.5 mg/kg, 0.09 mg/kg, and 0.010 mg/kg, respectively. When the confidence interval of 95% was determined, the detection limits of magnesium, zinc, and copper in serum were 0.7 mg/kg, 0.11 mg/kg, and 0.016 mg/kg, respectively.

### 3.5. Method Precision

The method precision was the relative standard deviation of the measurement results of 6 bottles of human serum reference materials. The experiments were carried out in 2 consecutive days, with 3 bottles each day, and 3 parallel for each bottle. The results are shown in Table 2. The precision for magnesium, zinc, and copper in different concentrations of human serum reference materials was lower than 0.3%, 0.9%, and 0.6%, respectively.

### 3.6. Method Trueness

Magnesium, zinc, and copper in standard substances NIST956D, ERM-DA120a, and GBW09152 were analyzed by using isotope dilution mass spectrometry. Parallel analysis was conducted three times a day for three consecutive days, and the results were good, as shown in Table 3.

### 3.7. Uncertainty Evaluation

In this research, the uncertainty caused by factors such as the experimental reagents, the samples, the laboratory environments, the solution preparation, the instrument measurement, and the data processing has been evaluated as the source of uncertainty in the measurement process. It can be concluded that by evaluating the uncertainty of each parameter in formula (1), the uncertainty caused by each factor in the measurement process can be fully included. Each parameter in formula (1) is an independent parameter, and the uncertainty *u*_*c*_(*y*) related to measurement is calculated as follows:

(2) $$\begin{array}{c} u_{c}\left(y\right)=\sqrt{\overset{N}{\underset{i=1}{\sum }}{\left[\frac{\partial f}{\partial x_{i}}\right]}^{2}u^{2}\left(x_{i}\right).} \end{array}$$

The formula of the sensitivity coefficient (*∂f*/*∂x*_*i*_) of each parameter in formula (1) is as follows:

(3) $$\begin{array}{c} \frac{\partial C_{x}}{\partial C_{z}}=\frac{m_{Z_{c}}}{m_{Y_{c}}}\cdot \frac{m_{Y}}{m_{X}}\cdot \frac{R_{Y}-R_{B}}{R_{B}-R_{Z}}\cdot \frac{R_{Z}-R_{B_{c}}}{R_{B_{c}}-R_{Y}},\\ \frac{\partial C_{x}}{\partial m_{Y}}=C_{z}\cdot \frac{m_{Z_{c}}}{m_{Y_{c}}}\cdot \frac{1}{m_{X}}\cdot \frac{R_{Y}-R_{B}}{R_{B}-R_{Z}}\cdot \frac{R_{Z}-R_{B_{c}}}{R_{B_{c}}-R_{Y}},\\ \frac{\partial C_{x}}{\partial m_{x}}=C_{z}\cdot \frac{m_{Z_{c}}}{m_{Y_{c}}}\cdot \frac{-m_{Y}}{{m_{X}}^{2}}\cdot \frac{R_{Y}-R_{B}}{R_{B}-R_{Z}}\cdot \frac{R_{Z}-R_{B_{c}}}{R_{B_{c}}-R_{Y}},\\ \frac{\partial C_{x}}{\partial m_{Y_{c}}}=C_{z}\cdot \frac{{-m}_{Z_{c}}}{{m_{Y_{c}}}^{2}}\cdot \frac{m_{Y}}{m_{X}}\cdot \frac{R_{Y}-R_{B}}{R_{B}-R_{Z}}\cdot \frac{R_{Z}-R_{B_{c}}}{R_{B_{c}}-R_{Y}},\\ \frac{\partial C_{x}}{\partial m_{Z_{c}}}=C_{z}\cdot \frac{1}{m_{Y_{c}}}\cdot \frac{m_{Y}}{m_{X}}\cdot \frac{R_{Y}-R_{B}}{R_{B}-R_{Z}}\cdot \frac{R_{Z}-R_{B_{c}}}{R_{B_{c}}-R_{Y}},\\ \frac{\partial C_{x}}{\partial R_{z}}=C_{z}\cdot \frac{m_{Z_{c}}}{m_{Y_{c}}}\cdot \frac{m_{Y}}{m_{X}}\cdot \left[\frac{R_{Y}-R_{B}}{{\left(R_{B}-R_{Z}\right)}^{2}}\frac{R_{Z}-R_{B_{c}}}{R_{B_{c}}-R_{Y}}+\frac{R_{Y}-R_{B}}{R_{B}-R_{Z}}\cdot \frac{1}{R_{B_{c}}-R_{Y}}\right],\\ \frac{\partial C_{x}}{\partial R_{Y}}=C_{z}\cdot \frac{m_{Z_{c}}}{m_{Y_{c}}}\cdot \frac{m_{Y}}{m_{X}}\cdot \frac{R_{Z}-R_{B_{c}}}{R_{B}-R_{Z}}\cdot \frac{\left(R_{B_{c}}-R_{Y}\right)+\left(R_{Y}-R_{B}\right)}{{\left(R_{B_{c}}-R_{Y}\right)}^{2}},\\ \frac{\partial C_{x}}{\partial R_{B}}=C_{z}\cdot \frac{m_{Z_{c}}}{m_{Y_{c}}}\cdot \frac{m_{Y}}{m_{X}}\cdot \frac{R_{Z}-R_{B_{c}}}{R_{B_{c}}-R_{Y}}\cdot \frac{-\left(R_{B}-R_{Z}\right)-\left(R_{Y}-R_{B}\right)}{{\left(R_{B}-R_{Z}\right)}^{2}},\\ \frac{\partial C_{x}}{\partial R_{B_{c}}}=C_{z}\cdot \frac{m_{Z_{c}}}{m_{Y_{c}}}\cdot \frac{m_{Y}}{m_{X}}\cdot \frac{R_{Y}-R_{B}}{R_{B}-R_{Z}}\cdot \frac{-\left(R_{B_{c}}-R_{Y}\right)-\left(R_{Z}-R_{B_{c}}\right)}{{\left(R_{B_{c}}-R_{Y}\right)}^{2}},\\ \frac{\partial C_{x}}{\partial C_{B}}=-1. \end{array}$$

Therefore,

(4) $$\begin{array}{c} u_{c}\left(y\right)=\sqrt{{\left(\frac{\partial C_{x}}{\partial C_{z}}\right)}^{2}\cdot {\left(u_{c}\left(Cz\right)\right)}^{2}+{\left(\frac{\partial C_{x}}{\partial m_{Y}}\right)}^{2}\cdot {\left(u_{c}\left(m_{Y}\right)\right)}^{2}+\ldots +{\left(\frac{\partial C_{x}}{\partial C_{B}}\right)}^{2}\cdot {\left(u_{c}\left(C_{B}\right)\right)}^{2}}. \end{array}$$

This research has evaluated the uncertainty of measurement and Table 4 is the source of uncertainty for measuring the parameters. The uncertainty of the measurement of Type A is the experimental standard deviation of the 6 repeated instrument measurements, taking the worst result in the experiment as the evaluation data. The uncertainty of the measurement of Type B is the uncertainty of solution preparation, which is synthesized from the uncertainty resulting from the electronic balance calibration and the uncertainty resulting from weighing.

## 4. Conclusions

This isotope dilution mass spectrometry established in our laboratory with the quality of easy operation, high trueness, and good reproducibility was used to accurately analyze the contents of magnesium, zinc, and copper in human serum. In the isotope dilution mass spectrometry determination process, there is no need to measure the accurate amount of isotope, the enriched isotope, which reduces the requirements for mass spectrometry instruments [26, 27]. This method is suitable as a reference method for assigning values of magnesium, zinc, and copper in human serum.

## Figures and Tables

**Table 1:** Instrument parameters.

Parameter	Mg	Cu	Zn
ICP RF power (W)	1100	1100	1100
Gas flows (L/min)	0.97	0.97	0.97
Lens voltages (V)	6.75	6.75	6.75
Analog stage voltage (V)	−1700	−1700	−1700
Pulse stage voltage (V)	900	900	900
Dwell time per AMU	2 ms	2 ms	2 ms
Scan mode	Peak hopping	Peak hopping	Peak hopping
Sweeps/reading	25	25	25
Readings/replicate	15	20	25
Replicates	15	20	25
Cell Gas A: CH_4_ (mL/min)	1.4	0	0.45
RP q: Ar (mL/min)	0.8	0.25	0.25

**Table 2:** Repeatability of magnesium, zinc, and copper in human serum by ID ICP-MS.

		Mg (mg·kg^−1^)			Zn (mg·kg^−1^)			Cu (mg·kg^−1^)	
		Level 1	Level 2	Level 3	Level 1	Level 2	Level 3	Level 1	Level 2
Day 1	1^#^	18.0	23.3	28.7	0.625	0.831	1.597	1.06	1.61
		18.0	23.3	28.7	0.627	0.834	1.583	1.06	1.62
		18.1	23.2	28.9	0.646	0.835	1.610	1.06	1.62
	2^#^	18.0	23.3	28.8	0.631	0.834	1.597	1.07	1.60
		18.0	23.3	28.8	0.627	0.825	1.594	1.06	1.62
		18.0	23.2	29.0	0.634	0.832	1.595	1.06	1.62
	3^#^	18.0	23.3	28.7	0.628	0.818	1.607	1.06	1.61
		18.0	23.3	28.7	0.634	0.819	1.599	1.06	1.63
		18.0	23.3	28.9	0.625	0.826	1.596	1.06	1.61

Day 2	4^#^	18.0	23.3	28.7	0.644	0.828	1.601	1.05	1.60
		18.0	23.2	28.7	0.634	0.827	1.606	1.05	1.60
		18.0	23.3	28.7	0.633	0.825	1.616	1.05	1.60
	5^#^	18.0	23.2	28.7	0.634	0.827	1.599	1.05	1.61
		18.0	23.3	28.7	0.632	0.830	1.596	1.06	1.60
		18.0	23.3	28.7	0.629	0.826	1.600	1.06	1.62
	6^#^	18.0	23.3	28.7	0.628	0.823	1.606	1.06	1.60
		18.0	23.2	28.7	0.633	0.830	1.591	1.06	1.59
		18.0	23.3	28.8	0.632	0.825	1.602	1.06	1.60

*s*		0.03	0.03	0.09	0.006	0.005	0.008	0.005	0.010

Avg		18.0	23.3	28.8	0.632	0.828	1.600	1.06	1.61

CV		0.2%	0.1%	0.3%	0.9%	0.6%	0.5%	0.4%	0.6%

**Table 3:** Analysis of standard reference material by two-way ID ICP-MS.

		Mg (mg·kg^−1^)				Zn (mg·kg^−1^)		Cu (mg·kg^−1^)	
		NIST956d			GBW	ERM	GBW	ERM	GBW
		Level 1	Level 2	Level 3	09152	-DA120a	09152	-DA120a	09152
Day 1	Test 1	34.98	22.91	10.49	20.59	0.649	1.156	1.126	1.064
	Test 2	34.84	22.70	10.38	20.54	0.664	1.148	1.131	1.069
	Test 3	34.60	22.72	10.41	20.61	0.660	1.140	1.126	1.073

Day 2	Test 1	35.08	22.51	10.37	20.56	0.653	1.140	1.145	1.082
	Test 2	34.60	22.95	10.43	20.63	0.652	1.156	1.140	1.077
	Test 3	34.66	22.83	10.51	20.52	0.650	1.144	1.143	1.080

Day 3	Test 1	34.74	22.59	10.36	20.50	0.665	1.147	1.131	1.069
	Test 2	34.67	22.66	10.25	20.60	0.658	1.137	1.130	1.067
	Test 3	35.04	22.73	10.28	20.56	0.651	1.156	1.135	1.072

Avg		34.80	22.73	10.39	20.57	0.656	1.147	1.13	1.072

cv		0.5%	0.6%	0.8%	0.2%	0.9%	0.7%	0.6%	0.6%

Certified values		34.96 ± 0.24	22.83 ± 0.16	10.30 ± 0.08	20.75 ± 0.44	0.658 ± 0.033	1.132 ± 0.056	1.130 ± 0.033	1.085 ± 0.044

Bias (%)		0.46%	0.42%	−0.84%	0.88%	0.33%	−1.35%	−0.37%	1.15%

**Table 4:** Sources of uncertainty in the determination.

Sources of uncertainty	Mg		Zn		Cu	
	GBW09152		GBW09152		GBW09152	
	Value (*x*_*i*_)	*u*_*c*_ (*x*_*i*_)	Value (*x*_*i*_)	*u*_*c*_ (*x*_*i*_)	Value (*x*_*i*_)	*u*_*c*_ (*x*_*i*_)
Type A uncertainties						
*R*_*Y*_	0.014	0.000	0.034	0.000	0.004	0.000
*R*_*z*_	6.667	0.021	6.208	0.014	2.107	0.003
*R*_Bc_	0.920	0.002	1.041	0.003	1.080	0.004
*R*_*B*_	0.885	0.003	1.004	0.003	1.049	0.002
*C*_*B*_ (mg·kg^−1^)	0.5	0.3	0.09	0.05	0.010	0.005
Type B uncertainties						
*C*_*z*_ (mg·kg^−1^)	19.398	0.003	1.290	0.002	1.008	0.002
*m*_zc_ (mg)	3.0 × 10^2^	0.040	2.6 × 10^2^	0.040	5.4 × 10^2^	0.040
*m*_Yc_ (mg)	5.5 × 10^2^	0.040	2.0 × 10^2^	0.040	4.0 × 10^2^	0.040
*m*_*Y*_ (mg)	3.4 × 10^2^	0.040	200 × 10^2^	0.040	4.0 × 10^2^	0.040
*m*_*x*_ (mg)	2.0 × 10^2^	0.040	300 × 10^2^	0.040	4.8 × 10^2^	0.040
Combined types A and B	—	0.25	—	0.045	—	0.011
Degrees of freedom (Veff)		8		8		8
Coverage factor (k)	—	2	—	2	—	2
Measured value (mg·kg^−1^)	20.57	—	1.147	—	1.072	—
Expanded uncertainty (*U*($\bar{x}$), *k* = 2) (mg·kg^−1^)	—	0.2	—	0.04	—	0.08

## Data Availability

The data used to support the findings of this study are included within the article.
